# Correspondence: Reply to ‘Phantom phonon localization in relaxors’

**DOI:** 10.1038/s41467-017-01396-5

**Published:** 2017-12-05

**Authors:** Michael E. Manley, Douglas L. Abernathy, John D. Budai

**Affiliations:** 1 Materials Science and Technology Division, Oak Ridge National Laboratory, Oak Ridge, TN 37831 USA; 2 Quantum Condensed Matter Division, Oak Ridge National Laboratory, Oak Ridge, TN 37831 USA

## Introduction

The Correspondence by Gehring et al.^1^ mistakes Anderson phonon localization for the concept of an atomic-scale local mode. An atomic-scale local mode refers to a single atom vibrating on its own within a crystal. Such a local mode will have an almost flat intensity profile, but this is not the same as phonon localization. Anderson localization is a wave interference effect in a disordered system that results in waves becoming spatially localized^2^. The length scale of the localized waves is set by the wavelength^2^, which is ~2 nm in this case. This larger length scale in real space means narrower intensity profiles in reciprocal space.

**Fig. 1:**
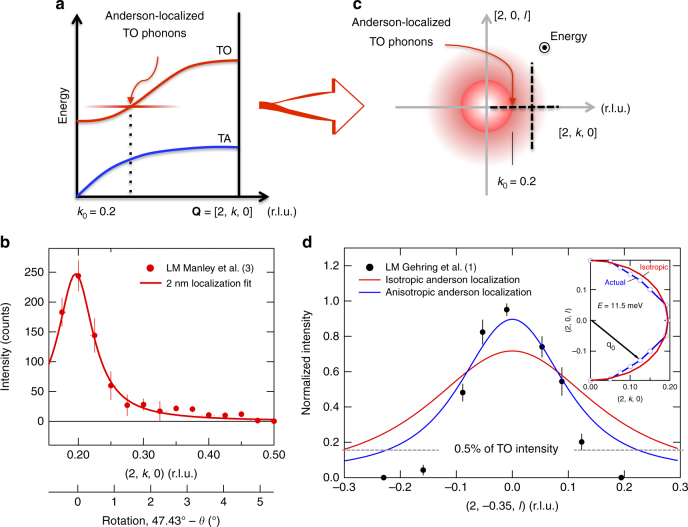
Analysis of the phonon localization structure in PMN-30%PT. **a** The structure of the Anderson-localized TO phonon in the high-symmetry direction appears dispersionless with an intensity maximum at the TO phonon, taken from our previous work^3^. **b** Measured local mode (LM) intensity profile along **Q** = [2, *k*, 0] with a 2 nm phonon localization fit, after our original work^3^. The extra axis at the bottom shows the crystal rotation angle *θ* for a more direct comparison with the tilt angle used by Gehring et al.^1^. **c** Projection of phonon localization profile in (2*kl*) plane assuming an isotropic TO phonon. In this view, energy points out of the page as indicated by the dot in circle. **d** Calculated phonon localization intensity profile compared with the tilting results of Gehring et al.^1^ ($l = 2.03 \times \sin ({\rm{tilt}}\,{\rm{angle}})$). The inset shows the measured anisotropy in the TO phonon, which was obtained from our original data^3^. The lowest data points corresponding to LM intensities less than ~0.5% of the TO phonon are likely below the detection limit of the instrument. In our measurements on BT7 the TO phonon peak counts^3^ of around 200 were about equal to the background intensity, so *N*_ph_ ~ *N*_bkg_ = *N*. The statistical noise in the background is $\sqrt N$ and a small peak with a fraction, *f*, of the phonon will have counts *fN*_ph_ ~ *fN*. At the detection limit, this gives *N* ~ 1/*f* ^2^. For *f* = 0.1%, *N* > 1,000,000 counts are needed, which requires counting more than 1000 times longer than we did

As described in our original article, because Anderson localization is exponential in real space^2^, it appears Lorentzian in reciprocal space^3^. Figure 1a illustrates the phonon localization structure along **Q** = [2, *k*, 0], after our original paper^3^. Figure 1b shows the measured profile along this direction in both reduced lattice units and crystal rotation angle. The localization-profile width, which corresponds to a 2 nm coherence length^3^, translates to a full width at half maximum of about 1° of crystal rotation. Gehring et al.^1^, however, tilted the crystal out of the plane along **Q** ≈ [2, 0.35, *l*]. Figure 1c shows how the Anderson localized phonon is expected to appear in the (2*kl*) plane assuming an isotropic TO phonon. Using an isotropic model, we calculated the intensity profile for the Gehring path based on our original fit shown in Fig. 1b. As shown in Fig. 1d, this isotropic model captures the basic shape of the intensity profile. An inspection of the TO phonon in our data shows that there is some anisotropy, and accounting for this results in better agreement, Fig. 1d. This analysis does not account for deviations from a vertical path in tilting, or the temperature difference (420 versus 488 K). The 420 K temperature used by Gehring et al. is a concern because it is only ≈10 K above the intermediate tetragonal phase, in which case they may have a mix of phases. They also assume an unrealistic lower limit of detection on the LM intensity. Nevertheless, the measured results of Gehring et al. are consistent with phonon localization at a length scale of ≈2 nm, and with our measurements^3^.

Addressing the second issue mentioned by Gehring et al.^1^, multiple scattering processes involving Bragg scattering and phonons (called ghostons^4^) cannot explain the phonon localization features. We previously ruled out multiple scattering using several arguments^3^. First, there is the strong temperature dependence of the intensity profile in the high-temperature phase (original Supplementary Fig. 6^3^)—which is unexpected for the ghostons since neither the underlying phonons nor Bragg peaks have significant temperature dependence. Second, there is the agreement between triple-axis and time-of-flight neutron scattering measurements—which employ different scattering geometries with quite different multiple scattering conditions (original Supplementary Fig. 3^3^). Third, the scattering symmetry shows that the LM appears the same across different zones, which correspond to different scattering conditions (original Supplementary Fig. 2^3^, and Fig. 1 in our more recent work^5^). Fourth, we modeled possible multiple scattering paths and could not identify a combination of Bragg plus phonon scattering that can fit the experimental results. Finally, the Bragg scattering intensities for our crystal were far too weak to support ghostons at the phonon localization intensities. The average diffracted beam was only about 0.2% the intensity of the incident beam. At its strongest point the phonon localization intensity reaches 30% of the TO phonon, which is 150 times too strong to be explained by ghostons.

We conclude that the claims in the Correspondence by Gehring et al.^1^ are incorrect because they mistakenly assume that the length scale for Anderson localization is atomic, and because the experimental observations rule out multiple scattering as the origin.

### Data availability

The data are available from the corresponding author on request.
